# Impact of a Psychoeducation on Caregiver Burden, Internalized Stigma, Anxiety, and Coping in Caregivers of Children With Epilepsy: A Randomized Pilot Study

**DOI:** 10.1111/nhs.70095

**Published:** 2025-05-12

**Authors:** Havva Kaçan, Halis Sakız

**Affiliations:** ^1^ Faculty of Health Sciences, Department of Nursing Kastamonu University Kastamonu Turkey; ^2^ Department of Educational Sciences, Faculty of Letters Mardin Artuklu University Mardin Turkey

**Keywords:** anxiety, caregiving burden, coping strategies, epilepsy, internalized stigma, psychoeducation

## Abstract

This pilot randomized trial examined the effects of a psychoeducational intervention on caregiving burden, anxiety, internalized stigma, and coping strategies among caregivers of children with epilepsy. Using a randomized design, 54 caregivers were assigned to either an experimental (*n* = 28) or control (*n* = 26) group. The 6‐week intervention included psychoeducation, emotional support, and stress‐management techniques aimed at enhancing caregivers' psychological well‐being. Results showed significant reductions in caregiving burden (*p* = 0.000), internalized stigma (*p* = 0.000), and anxiety (*p* = 0.000) in the experimental group, while the control group showed no significant changes. Caregivers in the experimental group also adopted more adaptive coping strategies, including increased self‐confidence (*p* = 0.000) and optimistic approaches (*p* = 0.001), greater reliance on social support (*p* = 0.001), and decreased use of helpless (*p* = 0.000) and submissive coping styles (*p* = 0.000). These findings demonstrate the feasibility and preliminary efficacy of psychoeducational interventions for improving caregiver well‐being and suggest the need for a future large‐scale definitive trial to confirm these effects.

**Summary**

- To the best of our knowledge, this is the first study conducted with parents regarding the epilepsy management of children with special needs in Türkiye.
- This study can provide a knowledge base for the evidence‐based practice of health professionals working for the health and well‐being of parents caring for children with special health needs.
- The results may help guide the development of similar programs in diverse cultural settings, addressing the specific needs of caregivers facing the stigma and stress of epilepsy care. By fostering an environment that encourages self‐confidence, optimism, and social support‐seeking behaviors, such interventions have the potential to enhance caregivers' well‐being and, in turn, positively influence the care provided to children with epilepsy.

## 1 Background

Chronic illnesses during childhood impose substantial physical, emotional, and psychological burdens on affected children and their families (González‐García et al. 2017). Chronic childhood diseases have a prevalence of approximately 10%–20% in the general population, with epilepsy, asthma, diabetes, and juvenile arthritis being among the most common (Er 2006; Meltzer et al. 2010). Epilepsy, in particular, is a prevalent neurological condition with significant impacts on a child's cognitive, social, and behavioral development (Wirrell et al. 2012; World Health Organization 2005). With a prevalence of around 0.5% in childhood, epilepsy most commonly manifests during early childhood or adolescence; approximately 50% of epilepsy cases begin before age 5 and 75% before age 20 (Fischer et al. 2022).

Epilepsy is often characterized by recurrent, unpredictable seizures caused by abnormal electrical discharges in the brain, with an incidence among children that varies between 41 and 187 cases per 100 000 (Camfield and Camfield 2015). The episodic nature of seizures, combined with potential comorbidities and a risk of sudden unexpected death in epilepsy, increases both the immediate and long‐term challenges for caregivers. Such complexities demand comprehensive management strategies that address not only the child's medical needs but also the family's emotional and psychological well‐being (Elliott et al. 2012; Kaye 2021).

The burden of caring for a child with epilepsy extends beyond medical care, affecting nearly every aspect of family life. Families with children diagnosed with epilepsy often face psychological challenges, including increased levels of stress, anxiety, and depression. Such emotional burdens are compounded by sleep disturbances, strained family relationships, and economic pressures due to additional caregiving responsibilities (Chang et al. 2021; Fazlıoğlu et al. 2010). Additionally, a diagnosis of epilepsy can also limit caregivers' employment opportunities, affecting financial stability (Hansen et al. 2018).

For families of children with special needs alongside epilepsy, the burden intensifies, as caregivers must navigate a web of medical appointments, therapeutic interventions, and social stigmas (Görkem and Bayram 2019). The demands of this care are well‐documented in the literature, with studies reporting that restricted social engagement, societal stigmatization, economic challenges, and lack of adequate information are recurring issues for caregivers (Aksoy and Demirli 2020; Gördeles Beşer and İnci 2014; Hill and Rose 2009; Şengül and Baykan 2013). Furthermore, caregiver burnout has been associated with negative outcomes for both caregivers and their children, indicating the importance of interventions to support their well‐being (Gona et al. 2011; Lee et al. 2022).

Caregivers of children with epilepsy frequently experience psychological distress as they manage the emotional toll and societal stigmatization associated with the condition (Fazlıoğlu et al. 2010). Studies highlight that anxiety and depression are prevalent among these caregivers, driven by uncertainties surrounding seizure occurrences, fear of injuries during seizures, concerns over medication side effects, and worries about social stigmatization (Gürhopur and Dalgiç 2018; Winsor et al. 2024). Research emphasizes that caregivers often experience social isolation, as the stigma associated with epilepsy discourages participation in community activities (DiIorio et al. 2006; Williams et al. 2003). Social stigma places a distinct psychological burden on caregivers. In some cultures, epilepsy is viewed with misconceptions that heighten feelings of shame and isolation for families (Fisher et al. 2014). However, stigma reduction interventions have shown promise in enhancing social support and decreasing caregiver isolation, yet further research is needed to assess their long‐term efficacy (Pepi et al. 2024; Taylor et al. 2011).

One promising approach to mitigating caregiver distress is psychoeducation, which has been shown to improve caregivers' emotional resilience and capacity to manage chronic childhood conditions. Psychoeducation provides structured information about the illness, its management, and coping strategies, empowering caregivers to navigate daily challenges more effectively (Arabacı et al. 2018; Gümüs et al. 2020; Lobban and Barrowclough 2009). For caregivers of children with epilepsy, psychoeducational programs have demonstrated potential in reducing fear, anxiety, and uncertainty surrounding seizure management while fostering adaptive coping strategies (Hagemann et al. 2016; Kaye 2021).

International studies suggest that psychoeducation can enhance caregivers' self‐efficacy, reduce anxiety, and decrease the stigma associated with epilepsy (Hessler et al. 2018; Nabbout et al. 2019; Sakız and Sarıçalı 2019). Training in seizure first‐aid and emergency response has been shown to improve caregivers' confidence in managing seizures, while broader psychoeducational programs provide emotional support and social connection (Kumar 2021; Oleas Rodríguez et al. 2024). However, despite these promising findings, few studies have specifically addressed the dual challenge of epilepsy and co‐occurring developmental disabilities in children. Research indicates that children with epilepsy are at increased risk of cognitive, behavioral, and emotional difficulties, often requiring additional support beyond seizure management (Reilly et al. 2014; Wirrell et al. 2012). These challenges are further amplified when epilepsy coexists with neurodevelopmental disorders such as autism spectrum disorder or intellectual disabilities, leading to greater caregiving burdens and heightened stress levels (Kaşak et al. 2023). Studies show that caregivers of children with both epilepsy and developmental disabilities experience more severe mental health impacts, social isolation, and financial strain compared to those caring for children with epilepsy alone (Camfield and Camfield 2015). Yet, existing research has primarily examined general caregiver burden, with limited exploration of how managing both conditions simultaneously compounds stress and caregiving demands (Chang et al. 2021; Lobban and Barrowclough 2009). Furthermore, there is a lack of psychoeducational programs specifically tailored to caregivers who must navigate both epilepsy‐related stigma and the additional complexities of developmental disabilities, despite evidence that such targeted interventions could improve caregiver well‐being and child outcomes (Batchelor and Taylor 2021; Gümüs et al. 2020; Hagemann et al. 2016).

This study addresses these gaps by implementing a structured psychoeducational intervention specifically designed for caregivers managing both epilepsy and developmental disabilities. By equipping caregivers with targeted knowledge and skills, the intervention aims to alleviate caregiver burden, improve coping mechanisms, and enhance their overall well‐being. Given the limited research on psychoeducational programs tailored to this population, this study serves as a randomized pilot trial to assess the feasibility, acceptability, and preliminary effectiveness of the intervention. The findings will provide critical insights for refining the intervention and guiding the design of a future definitive trial with a larger and more diverse sample.

This study contributes to clinical practice and the broader caregiving literature by introducing a psychoeducational program that integrates medical and psychosocial support strategies. While epilepsy management often requires a combination of medical and behavioral interventions, caregivers frequently report insufficient access to tailored training and resources (Elliott et al. 2012; Turan et al. 2022). Prior studies have shown that psychoeducation fosters adaptive coping mechanisms in caregivers of children with chronic illnesses, enhancing resilience, and reducing burnout (Deniz et al. 2009; Papadopoulos et al. 2019; Pepi et al. 2024). However, no existing psychoeducational interventions specifically target caregivers dealing with epilepsy in conjunction with developmental disabilities. By offering structured training and emotional support, this intervention seeks to reduce caregiver burden and improve their ability to manage both epilepsy and developmental disabilities more effectively.

By providing evidence on the efficacy of psychoeducation, this study aims to inform healthcare professionals and policymakers about the importance of caregiver‐centered interventions. The results may help guide the development of similar programs in diverse cultural settings, addressing the specific needs of caregivers facing the stigma and stress of epilepsy care (DiIorio et al. 2006; Taylor et al. 2011).

This pilot trial aims to assess the feasibility and preliminary efficacy of a structured psychoeducational intervention for caregivers of children with epilepsy and developmental disabilities. Specifically, the study seeks to evaluate whether the intervention can alleviate caregiver burden, anxiety, and internalized stigma while fostering more adaptive coping strategies. Accordingly, the study tests the following hypotheses:

-
  Hypothesis 1 *Caregivers who participate in the psychoeducation will experience reduced burden, anxiety, and internalized stigma compared to those who do not participate*.
-
  Hypothesis 2 *Caregivers who undergo psychoeducation will show improved coping strategies from pre‐ to post‐intervention*.

## 2 Methods

### 2.1 Design

This study employs a randomized pilot trial with a pretest‐posttest design and a control group to assess the feasibility and preliminary effectiveness of a psychoeducational intervention for caregivers. Primary caregivers of children with special needs who have also been diagnosed with epilepsy were randomly assigned to either the experimental or control group using a concealed allocation process to minimize selection bias. An independent researcher conducted the allocation to ensure that group assignments were not influenced by researchers or participants. The intervention consisted of a structured 6‐week psychoeducational program designed to reduce caregiver burden, anxiety, and internalized stigma while promoting adaptive coping strategies. Feasibility was assessed based on recruitment and retention rates, as well as participant adherence to the program (Eldridge et al. 2016). Assessments were conducted at baseline and post‐intervention to measure changes in these outcomes, providing preliminary data to inform future large‐scale trials.

### 2.2 Participants and Sampling

The study participants are primary caregivers of children with epilepsy who are diagnosed with disabilities such as autism spectrum disorder, Down syndrome, intellectual disabilities, or physical disabilities. The children receive educational services in three private rehabilitation centers affiliated with the Ministry of National Education in Kastamonu Province, Türkiye. The recruitment period for participants spanned from October 1, 2023, to January 1, 2024.

Participants were randomly assigned to either the experimental or control group using a computer‐generated randomization sequence. An independent researcher, not involved in participant recruitment or intervention delivery, generated the allocation list using block randomization with a 1:1 ratio to ensure equal group sizes. The sequence was concealed in sealed, opaque envelopes, which were opened sequentially as participants were enrolled. As a result, 30 individuals were allocated to the experimental group and 30 to the control group, totaling 60 participants.

Eligibility criteria included caregivers between the ages of 18 and 65 who have no mental health disorders, adequate mental capacity to understand and comply with study procedures, and willingness to participate for the 6‐week duration of the psychoeducation. Additionally, a power analysis conducted using G*Power 3.1 software indicated that, with an effect size of 0.80 and a significance level of 0.05, a minimum of 23 participants per group would yield a test power of 0.95, resulting in a total target of 56 participants to account for potential dropout. This sample size was deemed sufficient for feasibility assessment and to inform future large‐scale trials.

Of the 60 initially assigned participants, 54 completed the study, while six participants (three from each group) were lost to follow‐up due to scheduling conflicts or withdrawal from the program. Table 1 presents the full demographic and baseline characteristics of all participants, including those lost to follow‐up. A comparison between completers and dropouts indicated no significant differences in demographic variables such as age, education level, or caregiving duration (*p >* 0.05), suggesting minimal risk of attrition bias. Sensitivity analyses were conducted using intention‐to‐treat principles to ensure the robustness of results despite missing data.

**TABLE 1:** Descriptive characteristics of the participants.

Variable	Category	Experimental		Control		*χ* ^*2*^	*p*
		*n*	*%*	*n*	*%*		
Relation to child	Mother	26	92.9	24	92.3	0.006	0.666
	Father	2	7.1	2	7.7		
Caregiving support	Spouse	14	50.0	9	34.6	1.375	0.503
	Family	3	10.7	3	11.5		
	None	11	39.3	14	53.8		
Marital status	Married	22	78.6	22	84.6	0.326	0.414
	Widowed	6	21.4	4	15.4		
Employment status	Employed	4	14.3	4	15.4	0.013	0.604
	Unemployed	24	85.7	22	84.6		
Perceived economic status	Good	7	25.0	5	19.2	3.495	0.174
	Moderate	18	64.3	21	80.8		
	Poor	3	10.7	0	0.0		
Child medication usage	Yes	15	53.6	13	50.0	0.069	0.504
	No	13	46.4	13	50.0		
Seizure management practice	No action	11	39.3	11	42.3	1.995	0.573
	Wait	10	35.7	6	23.1		
	Position airway open	7	25.0	8	30.8		
	Call ambulance	0	0.0	1	3.8		
Educational training	Yes	9	32.1	7	26.9	0.176	0.452
	No	19	67.9	19	73.1		
Child gender	Female	12	42.9	10	38.5	0.108	0.480
	Male	16	57.1	16	61.5		
Child diagnosis	Physical disability	2	7.1	2	7.7	15.591	0.029*
	Epilepsy	7	25.0	0	0.0		
	Autism	3	10.7	7	26.9		
	Cerebral palsy	3	10.7	1	3.8		
	Intellectual disability	12	42.9	11	42.3		
	Bipolar disorder	1	3.6	0	0.0		
	Spina bifida	0	0.0	2	7.7		
	Down syndrome	0	0.0	3	11.5		
Duration of epilepsy diagnosis	0–5 years	15	53.6	18	69.2	7.472	0.058
	6–10 years	4	14.3	7	26.9		
	11–15 years	1	3.6	0	0.0		
	16+ years	8	28.6	1	3.8		
Hospitalization	Yes	12	42.9	15	57.7	1.187	0.207
	No	16	57.1	11	42.3		
Most recent seizure	None	0	0.0	4	15.4	5.714	0.222
	Today	19	67.9	12	46.2		
	This week	3	10.7	3	11.5		
	This month	4	14.3	4	15.4		
	Several years ago	2	7.1	3	11.5		
Seizure frequency	Daily	19	67.9	19	73.1	1.070	0.784
	Weekly	2	7.1	1	3.8		
	Monthly	3	10.7	4	15.4		
	Once every few years	4	14.3	2	7.7		
Treatment duration	0–5 years ago	10	35.7	6	23.1	3.766	0.288
	6–10 years ago	4	14.3	3	11.5		
	10–30 years ago	8	28.6	5	19.2		
	None	6	21.4	12	46.2		
Age of first seizure	0–1 year	14	50.0	19	73.1	3.158	0.206
	2–6 years	11	39.3	6	23.1		
	10–20 years	3	10.7	1	3.8		
Thoughts on epilepsy	None	3	10.7	8	30.8	5.932	0.115
	Difficult and exhausting	14	50.0	12	46.2		
	Not a concern; manageable with education	6	21.4	1	3.8		
	Fear and anxiety	5	17.9	5	19.2		
Time for self care	Yes	18	64.3	16	61.5	0.044	0.529
	No	10	35.7	10	38.5		
		*M*	SD	*M*	SD	*t*	*p*
Parental age		45.140	8.872	41.580	10.462	1.354	0.182
Child age		17.360	9.433	13.650	6.286	1.684	0.094

* *p* < 0.05.

### 2.3 Design and Implementation of the Psychoeducation Program

The intervention consisted of a structured 6‐week psychoeducational program designed to reduce caregiver burden, anxiety, and internalized stigma while promoting adaptive coping strategies. Feasibility was assessed based on recruitment and retention rates, as well as participant adherence to the program. After trial commencement, minor adjustments were made to eligibility criteria to enhance participant recruitment; specifically, the initial age limit for caregivers (set at ≤ 60 years) was removed to allow the inclusion of older caregivers facing similar caregiving challenges. Additionally, flexibility in session scheduling was introduced in response to participant feedback, ensuring greater accessibility. Assessments were conducted at baseline and post‐intervention to measure changes in these outcomes, providing preliminary data to inform future large‐scale trials.

Prespecified criteria for determining whether to proceed with a definitive trial included recruitment feasibility (target enrollment achieved within the planned timeframe), retention rate (> 85% completion), and preliminary evidence of intervention effectiveness based on changes in caregiver burden, anxiety, and stigma scores. These metrics were established to guide future research planning and resource allocation.

The psychoeducational program for caregivers of children with epilepsy was developed by the researchers based on structured individual and group psychoeducation models, drawing from relevant literature and expertise (e.g., Gümüs et al. 2020). The content aimed to support caregivers in managing epilepsy‐related challenges and was validated by experts, including pediatric neurologists, child psychiatrists, and academic nurses.

Participants for the study were selected through randomization. Caregivers were contacted by the unit's responsible staff and secretary, who provided them with details about the study and invited them to the center for further information. Following initial recruitment, a pilot study was conducted with six participants under the supervision of the unit head. This pilot phase allowed for adjustments to the program based on initial feedback to ensure effectiveness and relevance to caregiver needs.

#### 2.3.1 Psychoeducation Sessions and Content

The psychoeducation program consisted of 6 weekly sessions, each lasting 45–60 min, conducted in a private room at the special education centers. Visual aids were created using Microsoft PowerPoint, and sessions included interactive elements and multimedia content tailored to the needs of caregivers. Sessions were structured as follows.

##### 2.3.1.1 Introduction and Epilepsy Basics

This opening module provided an overview of epilepsy, including types of seizures, causes, and symptoms, as well as its impact on caregivers and family members. The session aimed to build foundational knowledge and demystify misconceptions about epilepsy.

##### 2.3.1.2 Self‐Efficacy for Medication Management

Caregivers were educated on the importance of medication adherence, recognizing medication effects, and managing potential side effects. They learned strategies to support children in taking medications consistently and effectively.

##### 2.3.1.3 Seizure Management and Emergency Planning

This session included hands‐on training for managing seizures, emphasizing actions before, during, and after a seizure. Key skills included assessing consciousness, timing seizures, removing nearby hazards, loosening clothing, positioning the child safely (e.g., turning the head to the side), and calling for emergency assistance if needed. Simulation mannequins were used to practice these techniques, enhancing caregivers' preparedness for emergency situations.

##### 2.3.1.4 Anxiety and Stress Management

This session introduced relaxation and stress‐relief techniques to help caregivers manage anxiety associated with caregiving. Canva‐created videos demonstrated methods such as deep breathing, progressive muscle relaxation, and mindfulness. These videos were shared with caregivers on tablets for ongoing use at home, reinforcing skill application.

##### 2.3.1.5 Communication and Problem‐Solving Skills

Focused on enhancing caregiver‐child communication, this module covered effective interaction strategies and problem‐solving skills to navigate the unique challenges associated with caregiving for children with epilepsy. Activities and role‐playing exercises helped caregivers practice these skills in realistic scenarios.

##### 2.3.1.6 Reducing Stigma

The final module addressed societal stigma associated with epilepsy. Caregivers learned self‐advocacy strategies and resilience‐building techniques to navigate stigma and foster social inclusion. This session empowered caregivers to counteract discrimination and support their children's participation in community activities.

#### 2.3.2 Program Implementation

To accommodate caregivers' schedules, psychoeducation sessions were primarily conducted during working hours. However, adjustments were made to offer sessions outside of regular hours or on weekends for those with conflicting work or personal commitments. This flexibility was essential in ensuring sustained participation and program completion.

Caregivers in the control group did not receive the psychoeducational intervention. For both the experimental and control groups, pretest assessments were administered during an initial meeting at the center by the primary researcher, following an explanation of the study and the acquisition of informed consent. Posttest assessments were administered 8 weeks after the pretest, at the same location and by the same researcher, to evaluate the impact of the psychoeducational intervention.

The psychoeducational program content was reviewed by a panel of experts in pediatric neurology, child and adolescent psychiatry, and academic nursing. This review process ensured the material's accuracy, relevance, and appropriateness for the target audience, enhancing the program's overall credibility and effectiveness. Blinding was not feasible for participants and care providers due to the nature of the psychoeducational intervention. However, outcome assessors were blinded to group allocation to minimize bias in data collection. No harm to the participants was reported or observed.

### 2.4 Data Collection

Data were collected using several validated instruments with high reliability scores, applied as pre‐ and post‐tests to both groups. These tools include the following.

#### 2.4.1 Burden Interview

The Burden Interview was developed by Zarit et al. (1980) to assess the difficulties experienced by caregivers and document this situation. The Turkish adaptation, validity, and reliability of the scale, originally developed by Zarit and colleagues, were conducted by İnci (2006). The scale consists of a single subdimension. It includes 22 items and is administered without a time limit. The scale uses a Likert‐type response format ranging from “never” to “almost always,” scored from 0 to 4. Scores on the scale can range from a minimum of 0 to a maximum of 88, with higher scores indicating greater levels of difficulty experienced (Zarit and Zarit 1990). In this study, the reliability of the Caregiver Burden Scale was found to be Cronbach's *α* = 0.887.

#### 2.4.2 Parents' Internalized Stigma of Mental Illness Scale

Parents' Internalized Stigma of Mental Illness Scale, developed by Dikeç et al. (2020), was used in this study. The scale consists of 29 self‐report items using a four‐point Likert format. The scale includes five subscales: “Alienation” (items 1, 5, 8, 16, 17, 21), “Endorsement of Stereotypes” (items 26, 10, 18, 19, 23, 29), “Perceived Discrimination” (items 3, 15, 22, 25, 28), “Social Withdrawal” (items 4, 9, 11, 12, 13, 20), and “Resistance to Stigma” (items 7, 14, 24, 26, 27). Items are rated as “strongly disagree” (1 point), “disagree” (2 points), “agree” (3 points), and “strongly agree” (4 points). Items in the “Resistance to Stigma” subscale are reverse‐scored. The total scale score, obtained by summing the five subscales, ranges from 29 to 116, with no established cutoff score. Higher scores indicate a more severe negative impact of internalized stigma (Dikeç et al. 2020). In this study, the reliability of the scale was Cronbach's *α* = 0.874.

#### 2.4.3 Beck Anxiety Inventory (BAI)

The BAI, developed by Beck and Steer (1993), is a four‐point Likert‐type self‐assessment scale used to determine the frequency of anxiety symptoms experienced by individuals. The Turkish adaptation, validity, and reliability of the form were conducted by Ulusoy et al. (1998), with a Cronbach's *α* coefficient of 0.93. Total scores on the scale range from 0 to 63. Thirteen items assess physiological symptoms, five items explain cognitive aspects, and three items represent both somatic and cognitive symptoms (Ulusoy et al. 1998).

#### 2.4.4 Ways of Coping Questionnaire

The Ways of Coping Questionnaire was developed by Folkman and Lazarus (Folkman and Lazarus 1980) as a 30‐item, situation‐oriented measure designed to identify coping strategies used by individuals in general or specific stressful situations. The standardization study in Türkiye was conducted by Şahin and Durak (1995). The scale consists of five factors: Self‐confident (items 8, 10, 14, 16, 20, 23, 26), Helpless Styles (items 3, 7, 11, 19, 22, 25, 27, 28), Submissive (items 5, 13, 15, 17, 21, 24), Optimistic (items 2, 4, 6, 12, 18), and Seeking of Social Support (items 1, 9, 29, 30). Responses to the items range from “not suitable at all” to “very suitable” on a four‐point scale. No total score is obtained from the scale; separate scores are calculated for each subscale.

The reliability analyses of the scales used in the study were supported by high Cronbach's α values. For the Burden Interview, the reliability was calculated as *α* = 0.89 before the intervention and *α* = 0.92 after the intervention. Similarly, for the Parents' Internalized Stigma of Mental Illness Scale, reliability was found to be *α* = 0.88 before the intervention and *α* = 0.91 afterward. In the Back Anxiety Inventory, *α* = 0.87 was recorded before the intervention, increasing to *α* = 0.90 afterward. Within the Ways of Coping Questionnaire, the Self‐confident subscale demonstrated very high reliability with *α* = 0.86 before the intervention and *α* = 0.92 after. Similarly, for the Optimistic subscale, reliability was calculated as *α* = 0.85 before and *α* = 0.91 after the intervention. For the Helpless Styles subscale, reliability was *α* = 0.84 before and *α* = 0.88 after the intervention. The Submissive subscale showed consistent reliability, with *α* = 0.83 before and *α* = 0.87 after the intervention. Finally, the Seeking of Social Support subscale achieved high reliability levels, with *α* = 0.88 before and *α* = 0.93 after the intervention. These high Cronbach's *α* values indicate very high internal consistency and reliability for the scales used in the study.

### 2.5 Data Analysis

The data obtained in this study were analyzed using Statistical Package for Social Sciences (SPSS) for Windows, Version 22.0. Descriptive statistics—including frequencies, percentages, means, and standard deviations—were used to summarize the sample's demographic characteristics and baseline variables across scales.

To confirm the suitability of parametric tests, multiple normality assessments were conducted. Skewness and kurtosis values for all continuous variables fell within the acceptable range of ±1.5, as suggested by Tabachnick and Fidell (1996) and George and Mallery (2010), and within ±2.0 as a broader criterion for normal distribution (George and Mallery 2010). Additionally, Shapiro–Wilk (S–W) and Kolmogorov–Smirnov (K–S) tests were performed for each primary outcome measure. The Burden Interview showed no significant deviations from normality (K–S D(54) = 0.092, *p* = 0.200; S–W W(54) = 0.974, *p* = 0.210). Similarly, the Parents' Internalized Stigma of Mental Illness Scale met normality assumptions (K–S D(54) = 0.086, *p* = 0.200; S–W W(54) = 0.980, *p* = 0.270), as did the Beck Anxiety Inventory (K–S D(54) = 0.098, *p* = 0.170; S–W W(54) = 0.968, *p* = 0.150) and the Ways of Coping Questionnaire (K–S D(54) = 0.081, *p* = 0.200; S–W W(54) = 0.982, *p* = 0.290). Since all *p* values exceeded 0.05, none of the variables showed significant departures from normality. Furthermore, given the sample size (*n* = 54), the central limit theorem supports the use of parametric tests, as *t* tests remain robust to moderate violations of normality when *n* > 30 (Lumley et al. 2002; Schmider et al. 2010).

To compare categorical variables between the experimental and control groups, Chi‐square (*χ* ^2^) tests were applied, confirming no significant baseline differences in demographic and clinical characteristics that might confound the findings. For continuous variables, independent samples *t*‐tests were used to assess group differences post‐intervention, evaluating the impact of the psychoeducational program on caregiver burden, stigma, anxiety, and coping strategies. Within‐group comparisons were conducted using paired samples *t* tests to examine pre‐ and post‐intervention changes within each group. A two‐tailed significance level of *p* < 0.05 was adopted for all analyses.

### 2.6 Ethics

The study was approved by the ethics unit of a state university (approval number 9‐4, date 3.08.2023), with no external funding. Written permission was obtained from the institutions where the research was conducted. To ensure participant confidentiality, all data were anonymized, and no identifying information was collected. Each participant was assigned a unique code for data analysis. Informed consent forms outlined the voluntary nature of participation, the right to withdraw at any time, and the measures taken to protect personal data. All digital records were securely stored on a password‐protected system, accessible only to the research team, and will be deleted after the required retention period in accordance with ethical guidelines.

## 3 Results

Table 1 shows the descriptive characteristics of participants in both the experimental and control groups, with statistical analysis of differences between the groups on various demographic and caregiving variables. Below are key details for select variables.

The majority of participants in both groups were mothers, with no statistically significant difference in the relationship to the child, *χ* ^2^(1, *N* = 54) = 0.006, *p* = 0.666. Most were married, *χ* ^2^(1, *N* = 54) = 0.326, *p* = 0.414, unemployed, *χ* ^2^(1, *N* = 54) = 0.013, *p* = 0.604, and reported a moderate economic status, *χ* ^2^(2, *N* = 54) = 3.495, *p* = 0.174. The experimental group primarily received caregiving support from spouses or none, while the control group showed similar trends, *χ* ^2^(2, *N* = 54) = 1.375, *p* = 0.503.

In terms of epilepsy‐related factors, there were no significant differences between groups regarding medication usage, *χ* ^2^(1, *N* = 54) = 0.069, *p* = 0.504, seizure management practices, *χ* ^2^(3, *N* = 54) = 1.995, *p* = 0.573, or prior educational training on epilepsy, *χ* ^2^(1, *N* = 54) = 0.176, *p* = 0.452. Gender distribution of children, *χ* ^2^(1, *N* = 54) = 0.108, *p* = 0.480, hospitalization rates, *χ* ^2^(1, *N* = 54) = 1.187, *p* = 0.207, and most recent seizure, *χ* ^2^(4, *N* = 54) = 5.714, *p* = 0.222, were also similar across groups. Seizure frequency, *χ* ^2^(3, *N* = 54) = 1.070, *p* = 0.784, treatment duration, *χ* ^2^(3, *N* = 54) = 3.766, *p* = 0.288, and age at first seizure, *χ* ^2^(2, *N* = 54) = 3.158, *p* = 0.206, did not differ significantly.

The only significant difference observed was in child diagnosis, *χ* ^2^(7, *N* = 54) = 15.591, *p* = 0.029, with intellectual disability and epilepsy more common in the experimental group. Duration since epilepsy diagnosis approached significance, *χ* ^2^(3, *N* = 54) = 7.472, *p* = 0.058.

Parental and child ages were statistically comparable between the groups, *t*(52) = 1.354, *p* = 0.182; *t*(52) = 1.684, *p* = 0.094, respectively. Similarly, no significant differences were found in participant views on epilepsy, *χ* ^2^(3, *N* = 54) = 5.932, *p* = 0.115 or in their ability to find time for self‐care, *χ* ^2^(1, *N* = 54) = 0.044, *p* = 0.529.

Table 2 presents the descriptive statistics and *t* test results for caregiving burden, internalized stigma, anxiety, and coping strategies before and after the intervention. The table shows both the experimental and control groups' mean scores and standard deviations for each measure.

**TABLE 2:** Descriptive statistics and *t* test results for caregiving burden, internalized stigma, anxiety, and coping strategies.

Groups	Experimental (*n* = 28)		Control (*n* = 26)		*t* ^*a*^	*p*	Cohen's *d*
	*M*	SD	*M*	SD			
Caregiving burden pretest	40.357	10.545	40.539	10.760	−0.063	0.950	
Caregiving burden posttest	28.964	10.511	39.385	11.229	−3.522	0.001*	7.664
Within group (*t* ^*b*^)	7.664		1.786				
*p*	0.000*		0.086				
Cohen's *d*	0.017						
Internalized stigma pretest	54.500	12.069	51.154	9.748	1.115	0.270	
Internalized stigma posttest	41.929	8.734	51.231	9.697	−3.709	0.001*	5.996
Within group (*t* ^*b*^)	5.996		−1.000				
*p*	0.000*		0.327				
Cohen's *d*	0.304						
Anxiety pretest	23.357	6.308	23.692	11.048	−0.138	0.893	
Anxiety posttest	12.643	7.253	20.231	9.171	−3.385	0.001*	6.014
Within group (*t* ^*b*^)	6.014		1.527				
*p*	0.000*		0.139				
Cohen's *d*	0.038						
Self‐confident pretest	22.929	3.495	23.269	4.210	−0.324	0.747	
Self‐confident posttest	35.071	8.223	23.385	4.041	6.547	0.000*	8.710
Within group (*t* ^*b*^)	−8.710		−0.134				
*p*	0.000*		0.895				
Cohen's *d*	0.088						
Optimistic pretest	15.071	2.879	15.692	2.604	−0.829	0.411	
Optimistic posttest	22.536	2.411	15.500	2.789	9.937	0.000*	12.803
Within group (*t* ^*b*^)	−12.803		0.316				
*p*	0.000*		0.754				
Cohen's *d*	0.226						
Helpless styles pretest	16.036	3.977	15.692	3.631	0.331	0.742	
Helpless styles posttest	13.750	2.351	15.846	3.885	−2.418	0.022*	4.129
Within group (*t* ^*b*^)	4.129		−0.213				
*p*	0.000*		0.833				
Cohen's *d*	0.090						
Submissive pretest	17.679	3.486	17.808	3.633	−0.133	0.895	
Submissive posttest	15.964	3.144	18.115	3.525	−2.370	0.022*	3.959
Within group (*t* ^*b*^)	3.959		−0.489				
*p*	0.000*		0.629				
Cohen's *d*	0.036						
Seeking of social support pretest	12.000	2.293	11.769	2.197	0.377	0.708	
Seeking of social support posttest	20.429	3.458	12.154	2.203	10.395	0.000*	11.406
Within group (*t* ^*b*^)	−11.406		−0.670				
*p*	0.000*		0.509				
Cohen's *d*	0.103						

Abbreviations: *t* ^*a*^: independent samples *t* test; *t* ^*b*^: paired samples *t* test.

* *p <* 0.05.

In the experimental group, the mean Caregiving Burden score significantly decreased from 40.36 (SD = 10.55) before the intervention to 28.96 (SD = 10.51) after the intervention, *t*(27) = 7.66, *p* < 0.05, with a within‐group effect size of *d* = 0.017. The control group showed no significant change, *t*(25) = 1.78, *p* > 0.05. A significant difference was observed between the experimental and control groups after the intervention, *t*(52) = −3.52, *p* = 0.001, with a between‐group effect size of *d* = 7.664, indicating the intervention's effectiveness in reducing burden.

The experimental group showed a significant reduction in Internalized Stigma, with the mean score dropping from 54.50 (SD = 12.07) pre‐intervention to 41.93 (SD = 8.73) post‐intervention, *t*(27) = 5.99, *p* < 0.05, with a within‐group effect size of *d* = 0.304. The control group did not experience a significant change, *t*(25) = −1.00, *p* > 0.05. A significant difference was found between the groups post‐intervention, *t*(52) = −3.71, *p* = 0.001, with a between‐group effect size of *d* = 5.996, suggesting the intervention's positive effect on reducing internalized stigma.

In the experimental group, anxiety levels decreased from a mean of 23.36 (SD = 6.31) before the intervention to 12.64 (SD = 7.25) after the intervention, *t*(27) = 6.01, *p* < 0.05, with a within‐group effect size of *d* = 0.038. No significant change was observed in the control group's anxiety levels, *t*(25) = 1.52, *p* > 0.05. A significant difference was found between the groups post‐intervention, *t*(52) = −3.39, *p* = 0.001, with a between‐group effect size of *d* = 6.014, indicating the intervention's positive impact on reducing anxiety.

Regarding coping strategies, the experimental group showed a significant increase in Self‐Confident subscale scores from 22.93 (SD = 3.50) to 35.07 (SD = 8.22), *t*(27) = −8.71, *p* < 0.05, with a within‐group effect size of *d* = 0.088. The control group showed no significant change, *t*(25) = −0.13, *p* > 0.05. A significant difference between the groups post‐intervention was observed, *t*(52) = 6.55, *p* = 0.000, with a between‐group effect size of *d* = 8.710, indicating the intervention's effectiveness in fostering a self‐confident approach to coping.

Scores for the Optimistic subscale in the experimental group increased significantly from 15.07 (SD = 2.88) to 22.54 (SD = 2.41), *t*(27) = −12.80, *p* < 0.05, with a within‐group effect size of *d* = 0.226. No significant change was found in the control group, *t*(25) = 0.31, *p* > 0.05. A significant difference between the groups post‐intervention was observed, *t*(52) = 9.94, *p* = 0.000, with a between‐group effect size of *d* = 12.803, demonstrating the intervention's impact on promoting an optimistic approach.

The experimental group showed a significant decrease in the Helpless Styles subscale scores, from 16.04 (SD = 3.98) to 13.75 (SD = 2.35), *t*(27) = 4.13, *p* < 0.001, with a within‐group effect size of *d* = 0.090. The control group showed no significant change, *t*(25) = −0.21, *p* > 0.05. The groups differed significantly post‐intervention, *t*(52) = −2.42, *p* = 0.022, with a between‐group effect size of *d* = 4.129, suggesting the intervention helped reduce helpless coping strategies.

In the experimental group, the Submissive subscale score decreased from 17.68 (SD = 3.49) to 15.96 (SD = 3.14), *t*(27) = 3.96, *p* < 0.05, with a within‐group effect size of *d* = 0.036. No significant change occurred in the control group, *t*(25) = −0.48, *p* > 0.05. A significant difference between the groups post‐intervention was observed, *t*(52) = −2.37, *p* = 0.022, with a between‐group effect size of *d* = 3.959, indicating that the intervention helped reduce submissive coping strategies.

Finally, the experimental group showed a significant increase in the Seeking of Social Support subscale, with scores rising from 12.00 (SD = 2.29) to 20.43 (SD = 3.46), *t*(27) = −11.41, *p* < 0.001, with a within‐group effect size of *d* = 0.103. No significant change was observed in the control group, *t*(25) = −0.67, *p* > 0.05. A significant difference between the groups post‐intervention was observed, *t*(52) = 10.40, *p* = 0.001, with a between‐group effect size of *d* = 11.406, suggesting that the intervention was associated with increased social support‐seeking behaviors.

## 4 Discussion

### 4.1 Main Findings

The present study examined the effects of an intervention aimed at reducing caregiving burden, internalized stigma, anxiety, and maladaptive coping strategies while promoting adaptive coping mechanisms in caregivers of children with epilepsy. The findings demonstrate that the intervention was effective in significantly improving multiple aspects of caregivers' psychological well‐being, corroborating existing literature that highlights the benefits of psychoeducational programs in alleviating caregiver distress.

The significant reduction in caregiving burden observed in the experimental group following the intervention provides strong evidence for the effectiveness of psychoeducational programs in alleviating the strain experienced by caregivers of children with chronic conditions such as epilepsy (Gümüs et al. 2020). Prior to the intervention, both the experimental and control groups reported similar levels of caregiving burden, indicating that both groups initially faced comparable challenges in their caregiving roles. However, post‐intervention, the experimental group exhibited a significant decrease in caregiving burden, while the control group showed no significant change. This finding shows the effectiveness of the intervention in addressing the emotional and practical challenges that caregivers face.

The results align with research that has shown structured interventions, particularly those incorporating psychoeducation and emotional support, to significantly reduce caregiving burden in parents and caregivers of children with chronic health conditions like epilepsy (Aksoy and Demirli 2020). Caregiving burden, which includes physical, emotional, and financial strain, is a well‐documented concern for parents of children with epilepsy (Hansen et al. 2018). The intervention in this study likely provided caregivers with tools to manage the emotional and physical demands of caregiving, as well as the stress associated with unpredictable medical events such as seizures. This reduction in burden is especially important, as high levels of caregiving burden have been linked to adverse psychological outcomes such as depression, anxiety, and caregiver burnout (Lee et al. 2022).

This finding is consistent with previous studies that have shown psychoeducational interventions to be effective in reducing caregiving burden in similar populations. For example, Arabacı et al. (2018) and Kaçan and Sakız (2024) demonstrated that a psychoeducational program aimed at caregivers of children with chronic illnesses reduced both the emotional and physical burdens of caregiving. Similarly, Wright et al. (2024) found that caregivers who received education and emotional support reported lower levels of stress and caregiving burden, highlighting the importance of these interventions in improving caregivers' well‐being. These studies support the notion that providing caregivers with education, support, and coping strategies can significantly mitigate the stress and challenges associated with caregiving.

Additionally, the decrease in caregiving burden observed in this study may be linked to the reduction in anxiety and internalized stigma reported by the experimental group. As caregivers' anxiety decreased and their internalized stigma lessened, they may have felt more competent and less overwhelmed in their caregiving role. This reduction in emotional distress likely contributed to their ability to cope with caregiving challenges effectively, ultimately leading to a lower perceived burden. This connection between emotional well‐being and caregiving burden is consistent with research showing that interventions that address emotional and practical aspects of caregiving are effective in reducing burden (Arabacı et al. 2018).

The significant reduction in internalized stigma observed in the experimental group highlights the potential of psychoeducational interventions in addressing the emotional and psychological burden of caregivers. Internalized stigma, the process by which caregivers internalize negative societal attitudes about their child's condition, can profoundly affect mental health and overall well‐being. This aligns with the findings of studies that have examined how caregiving in the context of chronic health conditions like epilepsy can lead to internalized stigma and how interventions can mitigate this effect (Dikeç et al. 2020; Hansen et al. 2018; Sakız and Kaçan 2023).

The significant reduction in internalized stigma in the experimental group provides compelling evidence that psychoeducational interventions can help caregivers challenge and reframe these negative self‐beliefs. The intervention likely addressed internalized stigma by providing caregivers with psychoeducation about epilepsy and its management, which helped demystify the condition and reduce feelings of shame. By improving caregivers' knowledge and fostering a more accurate understanding of epilepsy, the intervention likely empowered them to reject stigmatizing beliefs and adopt a more positive and compassionate view of themselves and their caregiving role.

The reduction in internalized stigma found in this study is consistent with the findings of several studies that have shown psychoeducational interventions to be effective in decreasing stigma in caregiving populations. For instance, Sakız and Jencius (2024) demonstrated that interventions that challenge negative stereotypes and provide education about mental health conditions can significantly reduce internalized stigma. Papadopoulos et al. (2019) also found that caregivers who received education and support were less likely to internalize negative societal views and more likely to perceive caregiving as a meaningful and fulfilling role. These studies reinforce the idea that providing caregivers with the tools and support to challenge stigmatizing beliefs can have a profound impact on their mental health and overall caregiving experience.

The significant reduction in anxiety observed in the experimental group provides strong evidence supporting the effectiveness of psychoeducational interventions in improving mental health outcomes for caregivers. Anxiety is a pervasive concern for parents of children with epilepsy, as they frequently face the unpredictability of seizures and the constant worry about their child's health and well‐being (Williams et al. 2003). The findings of this study align with prior research that has consistently shown that caregivers of children with chronic health conditions, such as epilepsy, often experience elevated anxiety levels. However, these levels can be significantly reduced through targeted interventions that provide emotional support and coping strategies (Nabbout et al. 2019).

The anxiety reduction observed in the experimental group underscores the potential benefits of psychoeducational programs designed to address the unique emotional challenges faced by caregivers. One of the core components of the intervention involved teaching caregivers specific strategies to manage their anxiety. These included relaxation techniques, such as deep breathing or mindfulness, which have been shown to reduce anxiety in various caregiving contexts. Additionally, cognitive reframing—helping caregivers reframe their thoughts to view situations more positively or with less catastrophic thinking—could have contributed to this reduction. Cognitive reframing techniques have been particularly effective in reducing anxiety by altering caregivers' perceptions of stressful events (Orson and Larson 2021), such as seizures or hospital visits, and helping them manage feelings of helplessness.

These results are consistent with studies that have found that psychoeducational interventions can significantly improve caregivers' psychological well‐being. For example, Mercier and Dorris (2024) demonstrated that interventions focusing on emotional regulation and cognitive restructuring were effective in reducing anxiety and improving the mental health of caregivers of children with epilepsy. Similarly, Nabbout et al. (2019) found that caregivers who received support and training to manage their child's condition experienced less anxiety and greater emotional resilience.

In the broader context of caregiver research, anxiety reduction through psychoeducational interventions has been shown to have a profound impact not only on caregivers' mental health but also on their caregiving abilities. By reducing anxiety, caregivers are better able to think clearly, make informed decisions, and engage in positive caregiving behaviors, ultimately benefiting both their own well‐being and their child's health outcomes (González‐García et al. 2017). This supports the idea that addressing caregivers' emotional needs through targeted interventions can improve both the quality of care provided and the caregiver's overall life satisfaction.

The intervention had a profound effect on caregivers' coping strategies, which are central to managing the emotional and psychological demands of caregiving. In particular, caregivers in the experimental group demonstrated significant increases in self‐confident and optimistic approaches to coping, as well as a notable decrease in maladaptive strategies, such as desperate and submissive approaches. These findings highlight the importance of equipping caregivers with adaptive coping skills, which are crucial for improving both their mental health and caregiving effectiveness. The results are consistent with previous studies, which have highlighted the role of coping strategies in alleviating caregiver burden and enhancing well‐being (Skokan and Newman 2016).

The increase in self‐confidence and optimistic approaches to coping in the experimental group is a particularly encouraging finding. Self‐confidence is essential for caregivers, as it allows them to trust their abilities to manage caregiving challenges, which in turn can reduce feelings of helplessness and overwhelm. Similarly, optimism is a protective factor that helps caregivers maintain a positive outlook despite the challenges of caring for a child with a chronic condition like epilepsy. These adaptive coping strategies are critical for managing stress and preventing burnout (Görkem and Bayram 2019). The results of this study are consistent with research by Skokan and Newman (2016), which found that interventions that promote emotional regulation and cognitive restructuring can enhance adaptive coping strategies, such as self‐confidence and optimism. The intervention in this study likely provided caregivers with both the emotional support and cognitive tools needed to approach caregiving tasks with greater confidence and a more positive outlook.

Optimistic thinking has been shown to improve caregivers' ability to manage the stress and uncertainty associated with chronic illness (Nabbout et al. 2019). In this study, the intervention likely facilitated a shift in caregivers' cognitive appraisals of their caregiving roles, encouraging them to perceive challenges as manageable and within their control. These results align with Görkem and Bayram (2019), who found that caregivers with higher levels of optimism were better equipped to handle the stress of caregiving and exhibited better mental health outcomes.

In addition to the positive changes in self‐confident and optimistic coping strategies, the intervention also led to a significant reduction in maladaptive coping strategies, including the desperate and submissive approaches. Desperate coping, characterized by feelings of helplessness and attempts to escape the stress, can be emotionally exhausting and counterproductive, often leading to burnout and depression (Taylor et al. 2011). Submissive coping, where caregivers may feel powerless and overly deferential to others, can also exacerbate feelings of inadequacy and reduce caregivers' sense of agency (Şengül and Baykan 2013).

The reduction in these maladaptive strategies is particularly notable, as it suggests that the intervention helped caregivers replace these unhelpful responses with more adaptive ways of coping with the stress and challenges of caregiving. This aligns with findings from Oleas Rodríguez et al. (2024), who emphasized that educational programs, emotional support, and stress‐management tools can help caregivers transition from maladaptive to adaptive coping strategies. When caregivers are equipped with effective coping strategies, they are more likely to experience reduced stress and improved mental health outcomes (Skokan and Newman 2016).

The intervention also led to a significant increase in caregivers' use of social support. Social support is a well‐established protective factor against caregiving stress, as it provides emotional validation, practical assistance, and a sense of connection that can buffer the negative effects of caregiving (Siedlecki et al. 2023). The findings in this study confirm that caregivers who seek and receive social support are better equipped to manage the demands of caregiving, as social support has been shown to reduce feelings of isolation, lower anxiety, and improve overall emotional resilience (Taylor et al. 2011).

The experimental group's increase in social support‐seeking behaviors suggests that the intervention may be associated with a sense of community and belonging among caregivers within the study period. By encouraging caregivers to reach out for support, the intervention helped them recognize that they are not alone in their struggles and that support is available to them. This shift toward seeking support can significantly enhance caregivers' mental health by providing them with the necessary resources and emotional reinforcement to navigate the challenges of caregiving (Siedlecki et al. 2023). Wright et al. (2024) found that social support not only improves caregivers' emotional well‐being but also promotes adaptive coping behaviors, and the current study's results support this assertion. Overall, caregivers who feel confident and optimistic are better equipped to provide high‐quality care to their children with epilepsy, as they are less likely to become overwhelmed by the stresses of caregiving and more likely to engage in positive caregiving behaviors.

### 4.2 Strengths and Limitations of the Study

This study provides valuable insights into the potential benefits of psychoeducational interventions for caregivers of children with epilepsy. However, several limitations should be acknowledged. First, the relatively small sample size may limit the robustness of the findings. While the results are promising, future studies should include larger and more diverse populations to enhance statistical power and generalizability. Additionally, the study was conducted in a specific healthcare setting, which may restrict the applicability of the intervention to other populations with different socio‐cultural or healthcare backgrounds. Another key limitation is the reliance on self‐reported measures to assess caregiver burden, stigma, and coping strategies. Self‐reported data are susceptible to response bias, social desirability effects, and recall inaccuracies. Future studies could integrate objective measures, such as physiological indicators of stress (e.g., cortisol levels) or clinician‐reported assessments, to improve data reliability. Furthermore, this study did not include a follow‐up period, making it difficult to determine the long‐term sustainability of the intervention's effects. While the immediate post‐intervention results suggest improvements in caregivers' psychological well‐being, it remains uncertain whether these benefits persist over time or diminish once structured support ends. This raises concerns about potential relapse in caregiver burden, anxiety, and stigma. Future research should incorporate longitudinal designs with follow‐up assessments to evaluate the durability of these effects and identify factors influencing long‐term maintenance. Additionally, exploring the potential benefits of periodic booster sessions could provide insights into strategies for sustaining the intervention's impact over time. From a feasibility perspective, while this pilot study demonstrated that the intervention could be implemented successfully within the existing healthcare system, logistical challenges such as caregiver participation rates, attrition, and the availability of trained facilitators should be examined in future trials. Identifying potential barriers to participation and retention will be essential for refining the intervention before scaling it up for broader implementation.

### 4.3 Implications for Future Research

The findings of this study suggest that psychoeducational interventions may be an effective approach to enhancing caregivers' psychological well‐being. However, further research is needed to assess the intervention's applicability to different caregiver populations, healthcare settings, and cultural contexts. Future studies should focus on expanding the sample to include caregivers from diverse socioeconomic backgrounds to improve external validity. Longitudinal research is particularly important to determine whether the observed benefits persist beyond the immediate post‐intervention period. Follow‐up assessments at multiple time points (e.g., 6 months and 1 year) could help identify patterns of change in caregiver burden, anxiety, and stigma over time. Additionally, examining the role of individualized support versus group‐based interventions may provide insights into optimizing intervention delivery for different caregiver needs. To improve the practical implementation of psychoeducational interventions in clinical settings, future research should explore the cost‐effectiveness, scalability, and integration of such programs within routine healthcare services. Investigating the potential role of digital platforms or mobile health (mHealth) applications in delivering psychoeducational support could enhance accessibility, particularly for caregivers facing geographical or logistical barriers to in‐person participation.

## 5 Conclusion

This study provides empirical support for the effectiveness of a psychoeducational intervention in alleviating caregiver burden, reducing internalized stigma, and lowering anxiety levels among caregivers of children with epilepsy. The findings highlight the intervention's potential to promote adaptive coping strategies, ultimately enhancing caregivers' overall well‐being. While the immediate benefits are encouraging, the study also underscores the need for further research to evaluate the sustainability of these effects over time. In the context of future definitive trials, these results suggest that psychoeducational interventions should be tested in larger, more diverse populations with extended follow‐up periods. Additionally, refining intervention delivery methods and assessing cost‐effectiveness will be critical for ensuring broader applicability and integration into standard healthcare practices.

## 6 Relevance for Clinical Practice

Psychoeducational interventions designed for caregivers of children with epilepsy have the potential to improve not only caregivers' psychological well‐being but also the quality of care they provide to their children. By fostering self‐confidence, optimism, and social support‐seeking behaviors, these interventions can mitigate stress and enhance coping abilities. From a clinical perspective, integrating structured caregiver education programs into routine nursing and healthcare services could provide a sustainable approach to supporting families affected by childhood epilepsy. Future research should continue to explore the mechanisms underlying these improvements, the feasibility of implementing such interventions in different healthcare settings, and the potential for digital or hybrid formats to expand accessibility. Long‐term evaluations will be essential to determine whether booster sessions or supplementary interventions are necessary to sustain benefits over time.

## Author Contributions

**Havva Kaçan:** conceptualization, investigation, funding acquisition, methodology, data curation, supervision, resources, writing – original draft. **Halis Sakız:** conceptualization, writing – original draft, methodology, data curation, resources, writing – review and editing.

## Ethics Statement

Ethical approval for the research was attained from Kastamonu University.

## Consent

All participants were asked whether they consent to participate in the study. Those who consented could proceed to data collection.

## Conflicts of Interest

The authors declare no conflicts of interest.

## Supporting information


**Data S1.** Supporting Information.

**Data S2.** Supporting Information.

**Data S3.** Supporting Information.

## Data Availability

Data are available with this submission.
